# Memories of John Lisman

**DOI:** 10.3389/fncir.2018.00024

**Published:** 2018-03-27

**Authors:** Nonna A. Otmakhova, Nikolai Otmakhov, Leslie C. Griffith

**Affiliations:** Department of Biology, Brandeis University, Waltham, MA, United States

**Keywords:** phototransduction, CaMKII, Memory, oscillations, schizophrenia, large-scale integration

## Introduction

John Lisman had a passionate connection with life, art and science. He lived his life till the end with enthusiasm and energy, passing away on October 20, 2017. John said many times that he did not understand how people could retire from science—he personally would not know how to live without intellectually challenging himself every day. When he found out that he had cancer, and that his expected life span was “short or very short” he announced that he and his family were “not into grieving.” So it was business as usual. He just added the challenge of understanding cancer and treatment options to his list of challenges. He worked harder than ever, while making more time for his family and friends in the last months.

**Figure:**
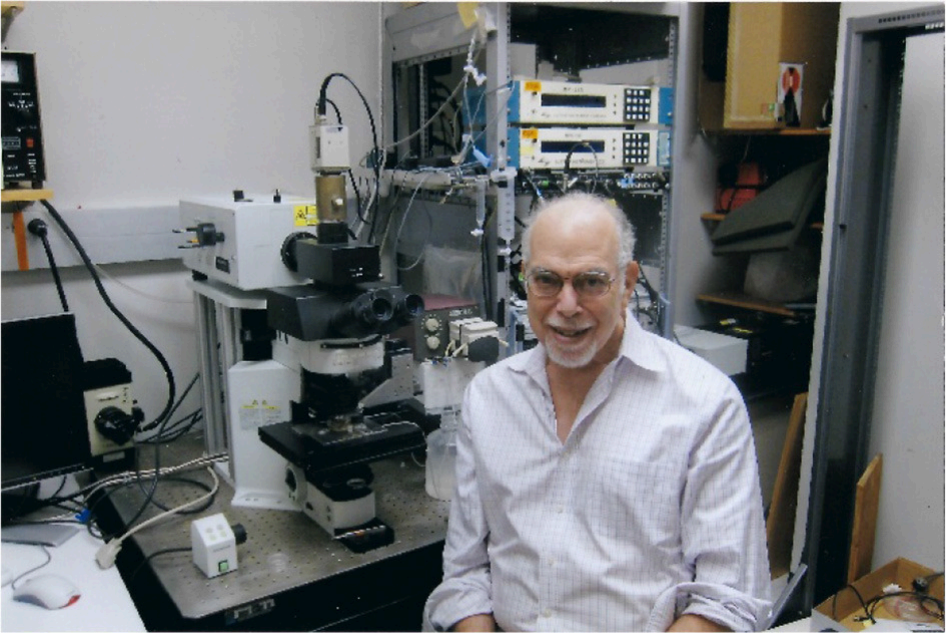


John's last lecture was delivered via Skype from his bed in the ICU at Sloan Kettering in New York City for the Brandeis Volen Retreat on October 12 to an audience largely consisting of his friends and Brandeis colleagues along with a few eminent guests. John had a life-long connection with Brandeis University. Brandeis was his home from the start of his academic career—he graduated cum laude with a bachelor's degree in physics in 1966—to the very end. He met his future wife here, mentored many students and postdocs and formed long-term collaborations with a significant number of his Brandeis colleagues. John's only academic time away from Brandeis was for graduate work at MIT and a postdoc at Harvard. At MIT with Joel Brown (1966–1971) he worked on unraveling cellular mechanisms of photoreception using the large photoreceptors of *Limulus*, combining electrophysiological measurements with mathematical modeling. After a short postdoc with Nobel laureate George Wald (1972–1974) he returned to Brandeis as an Assistant Professor, initially continuing his investigation of the mechanisms of *Limulus* photoreception but soon branching out into other areas and fields.

Mathematical modeling of biological processes was a staple of John's scientific approach. He believed that biology, like physics, should be driven by an idea, preferably in a mathematically described form. After returning to Brandeis he continued to work on *Limulus* phototransduction where he introduced many interesting ideas. Using all-custom-made equipment, his laboratory was able to record and characterize unitary light-dependent channels before any other sensory channels, providing a new direction for investigating the final steps of *Limulus* phototransduction. At the same time, he already began to think about applying his physicist's approach to other complicated biological problems. His first foray outside the field of phototransduction, which became a lasting obsession, was the molecular basis of memory. The initial idea grew from John's knowledge of rhodopsin acting as a phosphorylation-dependent molecular switch. In 1985 he published a model in which a group of kinases located at the synapse can phosphorylate each other, forming a bistable switch, a local molecular mechanism of memory storage. This type of molecular switch could be stably maintained in the face of protein turnover because individual molecules could be replaced by newly synthesized subunits which would be activated by neighbors. Concurrent work from Mary Kennedy's group showed that calcium/calmodulin-dependent protein kinase II (CaMKII) had properties that were similarly switch-like. Thus the CaMKII model of memory was born, driving both experimental and theoretical studies by many investigators.

As the CaMKII field developed, John continually modified his initial model, incorporating both new purely theoretical approaches (with a host of collaborators) and new experimental data from his lab and from the labs of colleagues. John's method for moving forward was to examine, discuss and synthesize. In his own lab group, models and data were ruthlessly dissected and debated to determine the direction of the next experiment or model. John would also engage in this process with pretty much anyone, anywhere, anytime. If he thought a person had an interesting point of view he would spend hours talking to them about CaMKII. John's last experimental paper on hippocampus-dependent spatial behavior and CaMKII was born of this process and was something he was very proud of—he considered it a final proof of the CaMKII model of memory.

A second area of interest for John was oscillations. This began with a purely theoretical project: the Lisman-Idiart 7 ± 2 model published in 1995. They proposed that the experimentally observed limit for human short-term memory capacity may be due to properties of oscillatory storage network. Each memory is temporarily stored in a separate high-frequency (gamma) subcycle nesting within a low-frequency (theta-alpha) oscillation and depends rather on changes in membrane excitability that on a reverberation mechanism. The model suggested that brain oscillations are a timing mechanism for serial processing of short-term memories. This paper became highly cited in the literature and created a number of new research directions in the Lisman lab and with collaborators. Another goal of this project was to understand how place-cell information is processed in different hippocampal sites based on oscillations and anatomical connections. Combining these ideas, John built a model of hippocampal functioning that he continued to refine based on new experimental data.

John's seemingly strange leap to the mechanisms of schizophrenia, a third area of focus, was catalyzed by his CaMKII research. John got interested in a role of dopamine in memory, postulating a linkage between D1 receptor-dependent inhibition of protein phosphatase 1 and CaMKII phosphorylation. In the process of experiments, new dopamine effects in the hippocampus were discovered that were unrelated to the CaMKII hypothesis. Combined with John's model of hippocampal function the dopamine effects led to some interesting ideas on the mechanism of schizophrenia. As was John's style, he intellectually engaged a broad range of other people in these efforts. Over the course of ~10 years, within the framework of a Conti Center grant, he hosted yearly immersion meetings for clinicians, experimenters and theoreticians, trying to create bridges between groups with different methods of studying schizophrenia. Initially these were centered on the role of the hippocampus, but later shifted to EEG pathology and the role of thalamus. While John felt that he and his collaborators had made inroads into understanding the genesis of the positive symptoms of the disease, he felt that new approaches were needed to understand the negative symptoms of schizophrenia. Among the last things he did was to initiate experiments to address this issue.

In this progression, it appears very logical that John would analyze and model attention and perception, ask what consciousness is, and what brain mechanisms are responsible for it. After publishing several papers on the subject, he secured a Kavli grant to organize a small meeting to do just that. In July 2017 John gathered ~10 scientists interested in large-scale integration in a big vacation house by the sea in Woods Hole, and they spent a week talking it over in a relaxed atmosphere.

This was John's genius. He was collaborating, discussing or arguing with half of the world. He wrote and received hundreds of e-mails in a day, reviewed numerous papers and grants. He organized and moderated dozens of meetings, liked to travel and to teach. He did not like to take “no” for an answer which made him rather difficult to disagree with. He would relentlessly come at a person with new arguments even when they would rather stop. Whether he was actually right or not, he brought so much passion to a discussion that it was hard to stop thinking about the relevant issues even after he left. Though he has now left us more permanently, the scientific discussion and debate he so dearly loved will continue. John would be pleased.

## Additional resources:

1. John Lisman's Photo Gallery of Neuroscience Colleagues: https://johnlisman.smugmug.com/
2. John Lisman, Memorial Blog: http://blogs.brandeis.edu/science/2017/10/21/john-lisman-1944-2017/
3. John's last lecture at the Brandeis Volen Retreat, October 12, 2017: http://www.bio.brandeis.edu/Lisman_Oct_12.mp4
4. Obituaries: (Jensen and Idiart, 2017; Kepecs, 2018).

## Personal tributes and memories

### Early study of *Limulus* phototransduction

**John E. Dowling**, Gund Research Professor of Neurosciences, Harvard University, Cambridge, MA, USA.

I do not remember exactly when I first met John; it may very well have been when he was a graduate student with Joel Brown at MIT and working on horseshoe crab phototransduction. I had been recording regenerative responses from *Limulus* photoreceptors at the Marine Biological Laboratory (MBL) in Woods Hole in the summers of 1968 and 1969, and this was something John was much interested in.

In 1972, John joined George Wald's laboratory at Harvard as a postdoctoral fellow, and I had just returned to Harvard after seven years at Johns Hopkins. It was during this time, 1972–74, that I saw John frequently. We became good friends and remained so over the years. I last saw John in Woods Hole late this past summer.

John was always a pleasure to be with—fun, interesting and excited about what he was doing and what we were doing. On Fridays in the 1970s and 80s, our lab organized lunch seminars with guest speakers. John was always there during his time in the Wald lab and frequently for many years thereafter while at Brandeis and still interested in *Limulus* phototransduction and vision. During his time at Harvard, he also became great friends with Gordon Fain, one of my graduate students, who was recording from vertebrate photoreceptors in mudpuppies and toads He and Gordon eventually published a most interesting hypothesis on why photoreceptors degenerate in vitamin A deficiency and retinitis pigmentosa.

Both John and I loved spending summers in Woods Hole and we would see each other there frequently—at lectures at the MBL, at the Captain Kidd, which is reputed to have the longest bar on Cape Cod, or wherever. I was always invited to the Annual Pepose Vision Lecture at Brandeis in the early spring, and often to make remarks after the marvelous suppers in John's and Natasha's beautiful apartment overlooking the Charles River, Cambridge, and Boston. Those were wonderful and unforgettable evenings.

John will be very much missed by all of us. Woods Hole in the summer will not be the same for me without John's presence.

**Gordon L. Fain**, Professor, Department of Integrative Biology and Physiology, UCLA, Los Angeles, CA, USA.

John and I met when he was a postdoc with George Wald in the early 70's. John was George's only student, and although Paul Brown was in the lab and was good company, Paul didn't have a very clear idea of what John was doing. So John spent a lot of time in the Dowling lab, where I was a graduate student. John had just finished his PhD with Joel Brown at MIT, where he had been studying the role of calcium in transduction using the photoreceptors of the *Limulus* lateral eye. He was using *Limulus* because the photoreceptors were unusually large, and in those days the only way to voltage clamp a neuron was to use two electrodes. The *Limulus* photoreceptor was one of the few preparations that would accommodate penetration with two intracellular electrodes and that could be used routinely and successfully for voltage clamp.

John and I became immediate friends. Our labs were a short distance away, and we participated together in seminars and journal clubs. We had many scientific interests in common, but we also became the sort of friends who could talk to one another about anything in our lives. Our families became close and continued to see one another even after my wife and I left Boston for UCLA and Santa Monica. What I admired most about John was his freedom. Perhaps because he was an only child doted on by loving parents, John had the confidence to pursue almost anything that interested him. This confidence had a big role in the way he did science. In my sensory physiology book, I refer to Lisman's law: “You have to believe it in order to see it.” The Derridas and Lacans of this world might say, “See! Science is like any other human endeavor, pervaded by subjectivity and incapable of ultimate truth.” But they would be wrong.What John meant is that scientific discovery begins with an idea. We start with an expectation, which we then hope to prove or disprove. This sort of creativity is, I think, what drew most of us into science in the first place, and it was John's great strength.

John had many interesting ideas about phototransduction and continued to work on *Limulus* into the new millennium. We collaborated on several reviews and read one another's papers. When John began working on memory in the mid-eighties, he still sent me his papers even though he knew that I wasn't familiar with the literature. I think he wanted me to know what he was doing and perhaps even try to seduce me into his field, but I was having too much fun working on rods and cones. Although there were clear advantages to John's *Limulus* preparation, there was no biochemistry, no genetics, and little prospect of obtaining either. As techniques improved and it became possible to record currents and voltage-clamp vertebrate photoreceptors, attention in the field drifted away from invertebrates. By that time John had very wisely moved his lab almost entirely into the CNS. I lost a valued colleague in my field, but we remained friends until the end of his life.

The cited review written with Richard Payne summarizes John's research on *Limulus* and gives a fairly clear picture of the state of *Limulus* phototransduction at about the time John left the field (Lisman et al., 2002b).

**Jay S. Pepose**, Professor of Clinical Ophthalmology and Visual Sciences, Washington University School of Medicine, St. Louis, MS, USA.

I worked with John Lisman in his lab at Brandeis University studying visual transduction in the ventral photoreceptors of *Limulus polyphemus*. Photoreceptors are nonlinear transducers, converting a large range of light intensities into a small range of steady-state voltages (the amplitude of the maintained receptor potential varies roughly as the logarithm of light intensity). We wondered what mechanisms might contribute to the photoreceptors responding in a graded fashion over a broad range of light energy. By using a voltage clamp technique and studying tail currents, we demonstrated the presence of voltage sensitive potassium channels. TEA was used to examine the functional role of the K+ channels because it blocks them without substantially affecting the light-activated Na+ conductance. The effect of TEA on response-intensity curves shows that the K+ channels serve to compress the voltage range of receptor potentials.

I am grateful to John's mentorship and friendship, which began when I was an undergraduate. This research resulted in my first publication, appearing in Pepose and Lisman (1978) and my first presentation at a scientific meeting—ARVO. His inquisitiveness was infectious and his influence served to launch my career in ophthalmology and visual sciences.

**Juan Bacigalupo**, Professor, Department of Biology, Faculty of Science, University of Chile, Chile.

A superior intelligence, passion, boundless insightfulness, curiosity and creativity, were John's most impressive characteristics. He was an enthusiastic explorer and a profound thinker with wide interests. This is what made John such a great scientist. His many fundamental contributions in a broad spectrum in the neurosciences, among which memory is the major protagonist, are well known and amply recognized. A representative example of John's approach to science is the amazing genesis of the idea of a molecular memory switch. The story began when he was a postdoc of George Wald (Nobel laureate) and continued at Brandeis. He was impressed by the fact that rhodopsin behaved as a molecular memory device, as it is turned on by light and stays activated until multiple phosphorylations turn it off. He published a series of papers with functional studies of rhodopsin, designing and conducting ingenious and thoughtful electrophysiological experiments on the horseshoe crab photoreceptors (*Limulus*). Much later, he became fascinated by the question of how memory is stored for undetermined lengths of time. He thought that the underlying mechanism had to reside locally at the thousands of dendritic synaptic spines that neurons can have. Inspired by rhodopsin, he reasoned that the fundamental basis for memory could be a protein that functions as a molecular switch. A holoenzyme with the ability to autophosphorylate that had recently been discovered in the postsynaptic density seemed perfect to account for his molecular switch. He conceived a powerful, simple and elegant theoretical model involving an autophosphorylating kinase that was turned on upon synaptic stimulation and that could dynamically overcome the problem posed by protein turnover to long-term memory storage. His paper with this model (Lisman, 1985) immediately positioned him among the leaders of the field, in which he pursued his investigations for the rest of his life.

For many years he carried out extensive investigations of a wide variety of aspects of phototransduction in the horseshoe crab, a favorite experimental model in the pre-patch clamp era, when electrophysiological experiments that could be performed on the tiny vertebrate photoreceptors were very limited. His contributions to this field were enormous. Using electrophysiology, he characterized in great detail the amazing physiological properties of these magnificent cells, whose unique large size allowed incomparable sophisticated studies with multiple microelectrodes. Their big single-photon responses gave relevant information about the early phases of phototransduction. The interplay of the different ionic conductances that gives shape to the light responses was thoroughly investigated. The multiple roles of calcium in this process were assessed with elegant experiments. The advent of the patch clamp technique allowed his laboratory to record and characterize unitary light-dependent channels, before any other sensory channel; these studies provided a new lead for investigating the final steps of *Limulus* phototransduction.

One aspect of John that few people may know was his boundless generosity, which I like to illustrate with my personal experience. I worked with John for about 12 years, starting in 1979 with my PhD thesis (I was his second graduate student) and ending when John abandoned his research on phototransduction to focus on memory. However, our close relationship continued until he died. Right after graduation I returned to my country to take a position at the University of Chile. At the time Chile was ruled by a repressive dictatorship and the economic situation was extremely difficult. Funding for science was almost inexistent. I was given a totally empty room to mount my laboratory, with no startup money whatsoever. In John's lab I had built the patch clamp set-up that I used in my thesis (characterization of the *Limulus* light-dependent channels), based on the famous patch clamp paper that had just come out (Hamill and Sakmann, 1981). I was hoping to get somehow the basic elements to build my set-up in Chile. However, the most critical piece of equipment that I was missing was a long working distance objective, which I could not afford. Two days before my departure and out of desperation, I decided to ask John if he would give me the objective that I had used. “Let me think on it”, he answered. The following morning he told me “Juan, have the lens, actually why don't you take the whole set-up with you to Chile.” I couldn't believe my ears, I was shocked. I packed the most essential parts and mounted a patch-clamp set-up in my laboratory. His donations and the exciting and productive annual summer stays in Woods Hole and Brandeis for the following years allowed me to survive scientifically. My wife Cecilia Vergara (PhD from Harvard) came along with me, also to work with John, and in the last three years we brought our babies. Every single time that I came back to his lab he would ask me “Juan, what else do you need for your lab?,” “An oscilloscope” (one of many examples), “lets go and get one for you,” and he took me to the store in his car. He continued being highly supportive and encouraging to me forever, helping me in many ways even though we stopped collaborating. For these reasons and for all that I learned from John, I am deeply indebted to him.

### CaMKII–a molecular memory switch

**Mark Bear**, Picower Professor of Neuroscience, Picower Institute for Learning and Memory, Massachuretts Institute of Technology, Cambridge, MA, USA.

As a scientist, John Lisman was, above all else, an idea guy. He loved ideas and loved discussing them with like-minded colleagues. I first met John over 30 years ago, when he came to give a seminar at Brown University where I had recently been hired as an assistant professor. I remember the scene quite vividly: We sat in an empty, dusty room that was to become my new lab. In the middle of the room were two chairs. John sat in one, and I in the other. Almost immediately, we were transported into a different dimension. John was excited, not only to share his ideas about autophosphorylating protein kinases as a repository of synaptic memory, but also to learn more about visual system plasticity and my ideas about homosynaptic long-term depression and the sliding modification threshold of the Bienenstock-Cooper-Munro (BCM) theory. It was clear that we were kindred spirits. We truly enjoyed each other's company, and over the ensuing decades we sought each other out on multiple occasions to bounce ideas around, usually over a nice dinner and a glass of wine. One tale John told particularly resonated with me. John's postdoctoral advisor George Wald once had admonished him that “John, to believe something, you must see it!” As the story went, John countered that no, to see something, you must believe it! Good ideas and well-formulated hypotheses can serve as a guiding light, and give one the strength to persevere when those with less conviction might give up.

In addition to the gift of John's warm company and enlightened conversation, he also made a measureable impact on my career by sending me his graduate student, Alfredo Kirkwood, to join me as my first postdoc. I recall John's evaluation of Alfredo as “someone he could recommend to a friend.” Thank you John. As a postdoc Alfredo set the standard for studies of LTP and LTD in the neocortex and experimentally validated some of the ideas John and I had discussed at our memorable first meeting. Alfredo has gone on to have a successful career as a professor at Johns Hopkins.

John was also committed to undergraduate education as was I. Recognizing the need for an undergraduate textbook in neuroscience, my Brown colleagues Barry Connors and Mike Paradiso and I teamed up to write the introductory book “Neuroscience: Exploring the Brain.” One feature of this book is a series of boxes called “Paths of Discovery” in which distinguished scientists briefly tell the story of how discoveries are made. John was the star of one of these, recalling how the idea came to him that autophosphorylating kinases could serve as memory storage devices. I reprint the text of this box, written in 2005, below.

John, thank you for the memories.

**A Memorable Walk on the Beach**, by John E. Lisman (2005) ^*^ (provided by Mark Bear).

My graduate work was in photoreceptor physiology with Joel Brown at MIT. I then did a postdoctoral fellowship with George Wald, who had recently won the Nobel Prize for the biochemical characterization of rhodopsin. I was fascinated with the problem of visual transduction and fully expected that I would spend my career on this topic. But about 10 years into my faculty position at Brandeis University, a series of events led me to become involved in what was for me the altogether new field of memory research.

My ongoing work on rhodopsin had led me to become interested in protein kinases. Rhodopsin is a molecular switch that is turned on by light. It had just been discovered that light also causes rhodopsin to become phosphorylated and that this contributes to turning rhodopsin back off. In the course of reading about kinases, I came across a curious fact: some kinases phosphorylate themselves, a process called autophosphorylation.

That year I was teaching the introductory course in neuroscience, which included a substantial section about memory. For years I had peripherally followed the memory field. Indeed, if one went to the meeting of the Society for Neuroscience, the most exciting sessions were those dealing with memory, particularly the work on Aplysia by Eric Kandel. But I also took note of the “new kid on the block” in memory research: the field of long-term potentiation in the vertebrate hippocampus. At the time, only a few labs were working on LTP, but they were making interesting discoveries. In particular, it was clear that hippocampal neurons contained thousands of dendritic spines, which were the sites of individual synapses. Remarkably, it appeared that each of these synapses could independently undergo LTP.

In the spring of 1984, just before teaching the memory component of my course, I attended the meeting of the Association for Research in Vision and Ophthalmology, at a wonderful beach resort in Sarasota, Florida. Between sessions, one could walk on the beach. Although there were ample visual distractions, my mind occasionally turned to science. Knowing that I would have to teach about memory when I returned to Brandeis, I contemplated my lecture. I was impressed Kandel's work, but the ideas he had developed for Aplysia just didn't seem applicable to LTP. He argued that learning was a form of differentiation; the secret to memory thus lay in gene control. What I couldn't understand is how gene control from the nucleus could apply differentially to each of the thousands of hippocampal synapses distributed over the dendritic tree. It would seem much more sensible for memory storage devices to be localized to each synapse. But this thought was heretical: if a storage device was in each spine, it couldn't be made of DNA and would probably be made of protein. But covalent modifications of proteins are unstable, and even if one could think of a stable modification, the protein itself would eventually disappear in the course of protein turnover. Everything would thus be forgotten. How could unstable molecular changes underlie stable memory?

My eureka moment occurred on this stroll along the beach. The core idea was that a group of autophosphorylating kinase molecules localized at a synapse could make a stable switch. During LTP induction, these molecules would become phosphorylated, and this would make them active. If a kinase molecule was dephosphorylated or replaced in the course of protein turnover, it could be phosphorylated by other members of the group. The switch could stay on, perhaps indefinitely, and this showed how unstable molecules could produce stable information storage.

At the time my first article was published, it was known that CaMKII was highly enriched at synapses. On exciting visits to two labs working on this enzyme, those of Jimmy Schwartz and Mary Kennedy, I learned of the newly discovered autophosphorylation properties of CaMKII. This suggested that CaMKII might operate as a switch along the lines suggested by my model. Now 20 years later, the central role of CaMKII in LTP is well established, and there is compelling support from mutant mice for the idea that autophosphorylation of the enzyme is also crucial for learning. The key question now is whether CaMKII is important in only the first phases of memory or whether it is required throughout the memory process. It will be exciting to see the answer to this question unfold.

^*^Mark Bear: A later draft of this text appeared in Neuroscience: Exploring the Brain, 3rd edition, by Mark F. Bear, Barry W. Connors, and Michael A. Paradiso. Lippincott, Williams and Wilkens, 2007.

**Thomas Soderling**, Professor Emeritus, Vollum Institute, Oregon Health and Science University, Portland, OR, USA.

With the death of John Lisman in November 2017, the neuroscience community lost a highly respected leader. I met John in the early 1990s at a neuroscience meeting where I spoke on calcium/calmodulin-dependent protein kinase II (CaMKII), the major focus of my laboratory at the Vollum Institute (not to be confused with the Volen Center). CaMKII was a favorite molecule of John's because its critical localization at glutamatergic synapses, its extremely complicated activation mechanisms, and its substrate specificity for AMPA-type ion channels lent it to numerous models of synaptic plasticity. With his analytical mind, John focused on formulating and testing molecular models of synaptic plasticity.

**Figure:**
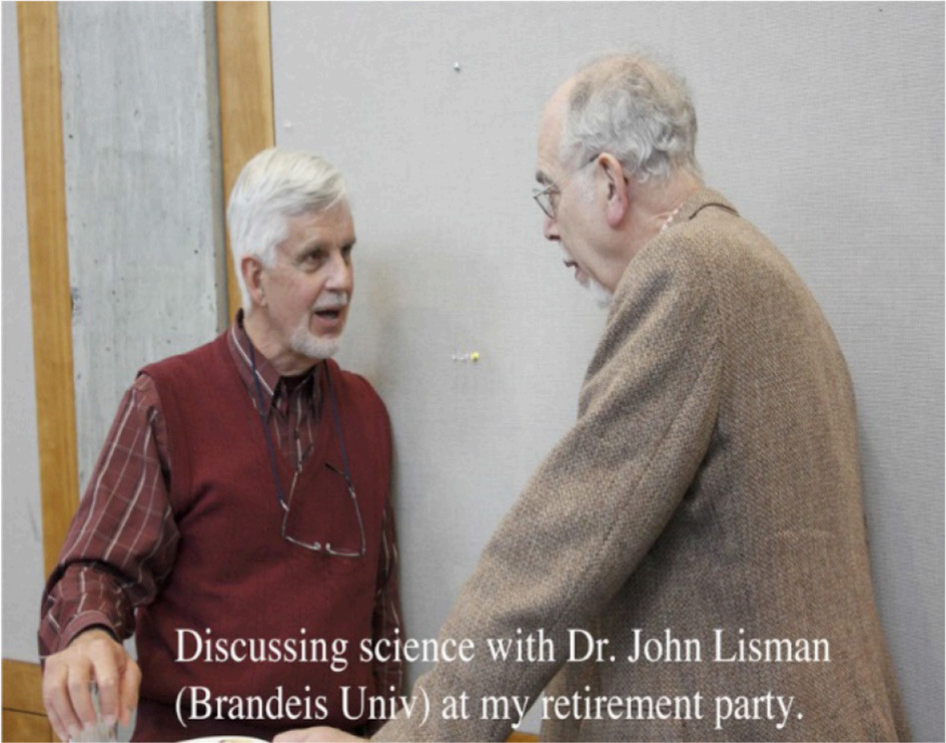


Whenever I heard John's voice on the other end of the phone, I knew I was in for at least an hour discussion of John's latest ideas on CaMKII. John was a frequent visitor to the Vollum Institute where he had many friends. John was a true friend of mine, a respected colleague, and he was a speaker at my retirement fest in 2012.

**Leslie C. Griffith**, Nancy Lurie Marks Professor of Neuroscience, Brandeis University, Waltham, MA, USA.

John was one of the first faculty members at Brandeis to seek me out and ask to speak with me when I arrived as an Assistant Professor in 1992. In retrospect, the topic of conversation was not surprising: CaMKII. Over the next 25 years we had many, many more conversations about CaMKII and even published a few papers together. Talking science with John was always intense, but fun. But John's intensity was not limited to science; it was just how he enjoyed life: art, food, wine etc. As many will attest, he could be an incredible amount of fun. John was a stimulating and challenging scientific colleague, but also a friend. I will miss him.

**Paul Miller**, Associate Professor of Biology, Brandeis University, Waltham, MA, USA.

John was kind, creative, enthusiastic, and hospitable. He was approachable, always willing to take the time to discuss and listen to ideas, however far from the beaten path, or to be supportive of a tearful student. Most Brandeis neuroscientists will have fond memories of him sharing the views over the Charles from his Penthouse suite in Watertown. Often the evenings—or days—in his apartment or his Woods Hole home would include after-dinner, pre-dinner, or multiple scientific talks. John's love for science meant it was never “work” that should be kept distinct from home comforts.

As a stalwart of the weekly journal club on computational and systems neuroscience, John's comments and contributions will be sorely missed. He would regularly attempt to cut through the technical details to address the key point of a paper to see whether it was a valuable contribution or not. While we discussed many of the projects that John was involved with, I worked with him most when attempting to put some rigorously calculated numbers into the question of just how stable a molecular switch—comprised of his beloved CaMKII coupled with a phosphatase—could be. Working with John was a lot of fun. When writing up the paper, I was impressed that he would edit and return each draft in less than a day, passionate as he was to get the story out. While that paper was in 2005, his enthusiasm and passion for the larger project remained with him till the end.

**Johannes W. Hell**, Professor of Pharmacology, UC Davis, Davis, CA, USA.

John was one of the very best scientists there are. He was deeply dedicated to understanding how our brain works. Perhaps one of his most remarkable characteristics was to develop such understanding without concerning himself whether he receives all the credit for it or not. John interacted with everybody who was interested in defining the molecular mechanisms of learning and memory and beyond. And he did so to his benefit. He never hesitated to adopt methods that were completely new to him. A great example is his recent publication in Neuron in which he used a sophisticated active avoidance task paired with viral injection to ectopically express kinase dead and constitutive active CaMKII. John called CaMKII a protein kinase with a memory or the memory protein because autophosphorylation kept CaMKII active beyond the initial Ca influx through NMDARs required for long-term potentiation and learning. In this publication he provided evidence for his model that CaMKII is required for maintenance of memory.

A few years earlier John realized that ectopic expression of the constitutively active CaMKII T286D mutant leads to autophosphorylation of CaMKII on T305 or T306, which suppresses Ca/CaM binding and thereby full activation of the kinase. This finding was most remarkable because John was by trait an electrophysiologist yet solved a puzzle with respect to earlier findings that CaMKII T286D did not behave as expected, i.e., purely as a constitutively active kinase, and did not simply augment synaptic strength. However, thanks to his inquisitive mind and openness to methods far beyond his original own ones he had developed a remarkably deep understanding of molecular mechanisms and how to analyze them.

John was a generous person who readily opened his home to visitors in Boston as well as at Woods Hole. Science would nearly always be the main point of discussions and I remember his wife Natasha state more than once “are you guys never getting tired of talking Science?” I hope John's mind has found the peace scientists like him never find in life because there is too much to explore.

**Nikolai Otmakhov**, Research Scientist II, Biology Dept., Brandeis University, Waltham, MA, USA.

I met John Lisman in 1993 during his summer collaboration with Roberto Malinow in Woods Hole. The goal was unimaginable for that time: optical quantal analysis from single dendritic spines in hippocampal slices. Our optical equipment was custom-made from parts and pieces and not quite up to the task; still we managed to establish the methodology and got promising results (Malinow et al., 1994). The most remarkable memory I have from that time is witnessing how John and Roberto discussed the project and planned experiments. They looked like two thrilled excited kids playing with building blocks. The process involved a lightning-speed exchange of facts and ideas, which was quite entertaining but also educational. It turned out that using custom equipment barely suitable to ambitious problems and approaching scientific goals with excitement and free-floating mind, were characteristic qualities for these two great scientists with whom I have had opportunity to work.

The early 1990s was the time when John transitioned from *Limulus* photo-transduction to the field of synaptic memory. His hypothesis that CaMKII works as a memory switch sustaining itself by self-activation due to autophosphorylation (Lisman, 1985, 1989; Lisman and Goldring, 1988a,b) just started gaining recognition. Several laboratories had already reported results consistent with the idea. There was one problem: initial attempts of the most critical experiment, which could prove or disprove the hypothesis, were contradictory.

In January 1994, I joined John's laboratory (small and underfunded at that time) and started to develop methodology for this critical experiment. The idea was simple: if a CaMKII activity-switch is persistently turned-on during memory formation, then inhibiting the kinase activity during memory storage should flip the switch back off to the resting state and reverse the memory. Importantly, the reversal should be permanent and not recover after removing the inhibition, while a new memory could be induced at that time, indicating that the memory switch had not been damaged (Lisman, 2017). We developed a methodology for reversible infusion of a CaMKII inhibitor intracellularly (through a patch-pipette) after induction of synaptic memory (long-term potentiation, LTP) in a single neuron. Simultaneous optical monitoring of the drug infusion/removal and electrical monitoring of synaptic strength (LTP) were also performed. The goal of finding out the mechanism of memory storage was thrilling and we worked hard to achieve it. After performing several modifications of the experiment with numerous controls, the data looked clean and convincing. To our huge disappointment, however, the results were not what we expected: the pre-established LTP was not reversed (Otmakhov et al., 1997; Chen et al., 2001). John seems to be the only one who was not discouraged: “There could be a number of reasons why the experiment failed.” He was strongly convinced of his hypothesis. “If an experiment does not confirm a theory, then there is a problem with the experiment”—that was his credo.

By that time, John already had published a new theoretical study (Lisman and Zhabotinsky, 2001) and reviews (Lisman, 1994, 2003; Lisman and McIntyre, 2001; Lisman et al., 2002a) in support of the CaMKII-switch idea and strongly believed in his theory. The reviews summarized existing publications, which in general were consistent with the role of the kinase in synaptic memory, but direct proof of the CaMKII-switch was still missing. In 1999–2004, a series of publications appeared from different labs showing that after activation and autophosphorylation, CaMKII can bind to synaptic NMDA receptors (NMDARs). This gave rise to a modified theory that the kinase plays a structural role in LTP (Otmakhov et al., 2004; Miller et al., 2005; Mullasseril et al., 2007). Again, the crucial observation of the persistence of this binding after LTP induction was missing. The experiment required new skills and new equipment, which we acquired.

The new series of experiments produced data consistent with the idea that CaMKII works as a structural seed that initiates and possibly maintains synaptic strength by controlling synaptic growth (Otmakhov et al., 2004; Asrican et al., 2007; Pi et al., 2010a,b; Feng et al., 2011; Otmakhov and Lisman, 2012; Lisman and Raghavachari, 2015). However, direct proof of this modified CaMKII-switch idea required a direct demonstration that the CaMKII-NMDAR complex persisted during the maintenance phase of LTP and behavioral memory and that disrupting the complex would reverse them both. Fortunately, a new CaMKII inhibitor (CN) had just been developed that could not only block the kinase activity but also interfered with the kinase binding to the NMDAR. Using this inhibitor Lisman's team with collaborators demonstrated results consistent with these predictions (Sanhueza et al., 2007, 2011).

Another breakthrough in advancing the CaMKII-switch theory was experimental confirmation from another lab that CaMKII holoenzymes (which consist of 10–12 subunits) could exchange their subunits in *in vitro* conditions. That provided potential proof for the second part of John's initial idea (Lisman and Goldring, 1988a), namely that a group of self-activating kinases can maintain its active conformation despite natural degradation/recycling of its individual units. So, John was determined to prove that the subunit exchange could indeed occur in living cells during the maintenance of LTP. The experiments of Sanhueza (Sanhueza et al., 2007, 2011), however, had two caveats: there was neither direct demonstration that the CaMKII-NMDAR binding occurs specifically in potentiated spines, nor that it persists into maintenance LTP phase.

To address these issues, we acquired a new technology that could directly measure both CaMKII activity in a single potentiated spine (Otmakhov et al., 2015a,b) and CaMKII binding to its spine targets like NMDAR (unpublished preliminary data). Furthermore, our lab also developed a behavioral methodology with viral intra-brain injections to prove that transient interference with either CaMKII activity and/or the kinase binding to its synaptic targets is critical for memory storage. During the past several years, John made heroic efforts to implement this last methodology just with the help of undergraduate students and produced compelling evidence for the role of CaMKII in the maintenance of behavioral memory (Rossetti et al., 2017). The molecular mechanisms of this involvement, however, are yet to be clarified. Although, several new studies were consistent with the core idea of Lisman's CaMKII memory switch through its autophosphorylation (Pi et al., 2010a,b; Zhang and Lisman, 2012; Kabakov and Lisman, 2015; Lisman and Raghavachari, 2015) direct evidence of this remains to be obtained. At his last lab meeting before his death, John suggested a very radical modification of his CaMKII-memory-switch hypothesis, which seemed to reconcile most of the existing conflicting data.

John's optimism, perseverance and unbreakable belief that he was right were unprecedented. He worked tirelessly on implementing new methodologies and skills, often with very limited resources. Shortly before his final days, he was awarded several grants to continue the course of studies, which should finally prove the involvement of CaMKII as a memory storage molecule. In parallel, he worked on a dozen other projects collaborating with numerous labs around the globe. He was on the phone or exchanging emails at all times of day or night. His optimism and enthusiasm were infectious and radiated confidence and encouragement. Despite this excitement, encouragement and patience, working with John was often quite frustrating. He was extremely biased, always finding reasons why conflicting data were incorrect; therefore, arguing with him was very challenging. Still, John's enthusiasm had been shaking up the neuroscience community for more than 30 years and to some degree shaped the course of research on the molecular memory storage and on many other fields of neuroscience as well. In 2017 alone, he co-authored more than 10 publications. What most impressed me in John's personality was his very sharp, dynamic and unpredictable mind. John could quickly grab a new set of data, dissect them at almost sub-molecular level and reassemble again, producing quite original and unpredictable hypotheses.

Despite his grave illness, John never intended to stop “playing” in science. His sudden passing in October 2017 was a shock for many people. For those of us who have worked with John for many years it is still difficult to accept his not being around. In addition to his optimism, John was quite realistic regarding his illness. Before his final days, he made sure that his research funding was transferred to collaborators and his life-long search for answers to the brain's mysteries continued.

### Dendrites, spines and synapses

**Kristen Harris**, Professor, Department of Neuroscience, Center for Learning and Memory, University of Texas, Austin, TX, USA.

I have known John since late Spring of 1986. I know this date because that was the only time that I accompanied my husband Max Snodderly to the Vision Meeting in Sarasota, FL. I attended that meeting because my son was a toddler, and I was afraid to stay home alone with him—really!

During that trip, I was mostly sitting on the beach under a cabana with our son, and 1 day, I noticed a rather large man lumbering down the beach toward our cabana. It was John Lisman, heading toward my little blue cabana. When he arrived, he plopped himself down in the sand, and said “Are you the ‘spiny lady’, Kristen Harris?” (Max had informed John of my whereabouts). I was baffled and honored that he would already know about my work—as the first few papers had barely come out. So, began our ongoing dialog about synapses and dendritic spines.

John and I wrote two opinion pieces (Lisman and Harris, 1993, 1994). Although these papers were written in the midst of the pre-post LTP “wars,” they have been well-regarded, and still receive citations. They only begin to describe the effects our conversations had on forcing us to think more clearly about the structural findings. In fact, just a few days before the recent Brandeis CaMKII symposium and John's untimely passing, John and I had exchanged a series of emails where he accused me of being “coy” because I wasn't ready to share some, as yet, unpublished data. The analysis was incomplete, and I had just 2 h to work before a pedicure appointment. I have been sorry, of course, that I did not share that data immediately, as I am quite sure John would have offered important insights.

This story emphasizes not only John's own great contributions to science, but his insatiable appetite for understanding other's work. His daughter Nora, at the celebration of John's life, relates how John would start his day at 1–2 a.m. reading over a cup of tea. A couple years ago, when John visited Austin, he stayed with Max and me. We too experienced John's awakening—as he began the morning session devouring a stack of papers he had brought with him. By 8:30 a.m., he was cat-napping on my office couch in preparation for his own fantastic talk at UT-Austin.

There is much to share about John, but one thing that keeps coming to my mind is the many visits with him at the MBL—where he would communicate his enthusiasm and synthesis of the latest literature over breakfast, lunch, and often dinner as well. Natasha and he often welcomed me to their wonderful home at MBL and there too he would invite other scientists to debate and discuss recent work from around the world. I once asked John how it was that he happily read so much of the scientific literature, every day. His response: “Oh, it is like a child waking up each morning and finding himself in a candy store—I just love science!! and the stories scientists tell.”

Recently, after mourning privately and on my face book page (“in the ether” as Natasha says) for a couple weeks, I finally had the energy to watch the beautiful celebration of John's life and to hear his final talk on CaMKII. I miss you John, but know that you are remembered—I believe it is in the memories of others that we live on as well as through the genes we pass on. John, you have succeeded wonderfully in both—creating our field's wonderful memories and producing such wonderful offspring, both biological and scientific. I too hope there is a consciousness that exists beyond our physical life, but even if not, know that the memories persist.

**Nelson Spruston**, Janelia Research Campus, Howard Hughes Medical Institute, Ashburn, VA, USA.

In the field of learning and memory, John Lisman ranks as one of the most influential thinkers of our era. In addition to his work on the role of CAMKII as a molecular switch mediating long-term synaptic plasticity, John thought deeply about how the nature of plasticity rules influenced the storage and recall of memories. I had the personal pleasure of having many long conversations with John about these issues; some of them were among the most memorable in my scientific career. I am grateful for the opportunities I had to spend time with John, both professionally and personally. He was a great colleague and friend.

**William Ross**, Professor of Physiology, Department of Physiology, New York Medical College, Valhalla, NY, USA.

I knew John for over 40 years. We first met in Woods Hole when he was working on *Limulus* photoreceptors and I was working on barnacle photoreceptors. We talked science but we soon became friends. Science always came first because he was a committed scientist. But we also shared other interests that deepened our relationship. After some years this friendship led to a collaboration with Dan Johnston using imaging to examine dendritic properties in hippocampal neurons—a productive time for all of us.

There are two things about John that were outstanding to me. The first was his inclusiveness as a scientist. He always had ideas and he wanted to share them. He would convene impromptu meetings of all sizes to discuss issues (often about some aspect of LTP or CaMKII, of course) and some of these would lead to collaborations. In the last few years we worked together trying to understand signaling in dendritic spines using sodium imaging. His ideas continue to inspire my work in this area.The second was his artistic temperament. He liked photography and his website of photo portraits of scientists is famous. His macro images of parts of the brain decorate several laboratories around the country. His everyday activities often had some creative aspect to them that gave flavor to the life around him.He was a free spirit in the sense that there was no boundary to the kinds of things he would think about. But more than that, he tried and often succeeded in making a contribution to each of these endeavors.

**William N. Green**, Professor, Department of Neurobiology, University of Chicago Chicago, IL, USA.

I got to know John Lisman through his association with the Marine Biological Laboratory (MBL) where for many years he was an inspiring and central figure in the MBL neuroscience community. John arrived at the MBL every summer from Brandeis University and would eagerly dive into all of activities that are part of MBL life. John's interests were incredibly broad and he was always willing to engage in friendly, collegial conversation. Most summers he would share a lab collaborating on different projects in the Whitman/Rowe Building. However, what he appeared to enjoy most was the time spent outside the lab in discussions and debate about his latest projects. My talks with John occurred in the lab, after seminars, at various social events and even on the Memorial Circle tennis courts where there was often more talk about science than tennis. At lunch, his discussions would continue through scheduled appointments at his lunch table out on the dock of the Captain Kidd. He was a fixture at all of the different weekly neuroscience seminars and often asked the most probing, perceptive questions. John was always very kind and generous with his time. Over the years, I began to rely more and more on John's advice and guidance especially as my scientific interests overlapped more with his. I will miss John's enviable ability to cut through the BS to get to the heart of a problem. It is hard to think about the MBL next summer without him. His loss leaves a big hole at the MBL where he will be sorely missed.

**Miquel Bosch**, Ph.D., Researcher, Institute for Bioengineering of Catalonia (IBEC), Barcelona, Spain.

I once asked John: “How on Earth are you able to keep track of new literature in the fields you are interested, which are pretty much ALL fields in Neuroscience?” His answer was: “I like reading.” And then he added “And writing. And thinking.” That conversation took place while walking rapidly through long corridors at Brandeis. At that time his long legs moved fast. As a new postdoc at MIT I was just delighted that the famous John Lisman had called me to come to his lab to discuss my new unpublished results. Yasunori Hayashi told him we had found that spines grew during LTP induction but that postsynaptic densities did not do it in synchrony, but with 1 h delay. I realized he could be discussing results and theories with young students, or postdocs, or famous senior scientists… with the same passion and respect; no distinctions; no hierarchies.

At another time, I asked John: “How are you able to propose so many theories, in some many different fields, and even to get to obtain enough experimental results to back them up?” His answer: “It is a tough job. It takes so many years to prove one single idea…” That conversation took place in Barcelona, this last spring of 2017. I enjoyed giving him a ride with my car through campus. His long legs were not that fast anymore and barely fit into my small car. But his brain was as fast as usual. He was proud of his last results demonstrating the central role of CaMKII in memory. “What else does this theory need to be accepted?” he was asking everyone in that congress.

Long live CaMKII and John's way of doing science.

**Valentin Nägerl**, Professor of Neuroscience and Bio-Imaging, University of Bordeaux, Bordeaux, France.

I had the pleasure and honor of sparring with John at various SfN meetings over the years; I remember how much he liked our poster on optical quantal analysis with calcium transients and spines the size of two A4 cardboards. When he showed up in the aisles at the SfN meeting, the mean IQ, age and kookiness would increase palpably! A couple of years ago he got a grant to revisit CaMKII in synaptic plasticity using super-resolution microscopy with us in Bordeaux. “Let's watch growing spines with STED induced by 2P uncaging and patch the cells at the same time, while single-molecule tracking of mutant forms of CaMKII”–“John, hold your horses”–“Listen, I don't have time to waste, we got to get to the bottom of things!” His great stamina paired with a child-like stubbornness and ironical wit—priceless and unforgettable! Here is to John, chapeau bas!

### Nesting oscillations for short-term memories

**Marco Idiart**, Professor, Institute of Physics, UFRGS, Porto Alegre, Brazil.

I first met John when I was a postdoc in the Department of Physics, at Brandeis in the early 90's. John was ubiquitous in all neurosciences events, which I eagerly attended in my transition from Statistical Physics to Computational Neuroscience. He was hard to miss, being a large man, always inquisitive and smart, but also very affable and soft-spoken. As I knew some people in his lab, I started visiting it even before officially meeting John. Eventually we ended up chatting about science and soon I was working for him in the newly built Volen Center for Complex Systems. The project resulted in the theta/gamma model for short-term memory published in 1995 in Science. I was very fortunate. The model has attained great recognition and is very influential in the area of brain oscillations, having received empirical support from numerous studies. The genesis of the model was marked with intense discussions with many scientists, later including Ole Jensen and Michael Kahana who helped to further develop and test it. I profoundly admired John's style of doing science, openly discussing ideas, not minding criticism but instead using them to push forward.

We were friends and collaborated for more than 20 years. Visiting John had become for me a vacation routine. Brazilian summer vacations are around January and I would always consider either visiting John in the inhospitable cold winter of Boston or going to a paradisiac beach in the north of Brazil, and often Boston would be the preferred choice. That was what my wife Aline and I did last January 2017. We spend almost a month with John working on a brain model for language in collaboration with our friend Boris Katz, from MIT. John being a renaissance man had planned in advance options for entertainment and good food. That was the last time I saw him in person.

After John found out about his illness in April 2017, we were in contact regularly. He would talk very openly about his grave health problems but I never saw him depressed or with low morale. He even had ideas for alternative treatments that he would challenge his doctors with. Most of the time we would talk about science and future plans. He was in the middle of an important referee battle and we had a paper in the making. He departed too soon and I will miss him dearly. He was a mentor, an inspiration and an extraordinary friend.

### Hippocampus-dependent spatial behavior

**Edvard Moser**, Kavli Institute for Systems Neuroscience, Norwegian University of Science and Technology, Trondheim, Norway.

I am shocked and saddened by John's premature passing. John was a good friend who visited me and the lab on many occasions. Until the very last weeks of his life, we had discussions about place and grid cells, and memory and space, and only weeks before he passed away we planned to meet at the Society for Neuroscience meeting. John was a true scientist. He asked and discussed until he understood, and new thoughts and questions popped up all the time, when we met or when he slept on it and suddenly woke up with a new idea, running to the computer and writing during very early morning hours. John helped us as examiner for several of our students. Discussions could be tough—reaching down to the bottom of the matter—but he was always friendly and there was always a smile. With his original approach, John has left long-lasting traces in the neuroscience community.

**César Rennó-Costa**, Digital Metropolis Institute, Federal University of Rio Grande do Norte, Brazil.

John's inaugural words to our partnership were: “I am curious about rate remapping.” To use curiosity as a professional drive and to cut to the chase were valuable lessons reinforced in many other opportunities. At that time, I was a freshman Ph.D. student in Barcelona and John was a lecturer at our annual summer school. My supervisor, Paul Verschure, introduced me as a technically-skilled student and John had the habit of trusting unknown junior scientists with his mental challenges. It was a perfect bond. He was very curious about a new form of brain code—rate remapping (Leutgeb et al., 2005)—and needed someone to help him model it. Even though he barely knew me, after a 5-min chat he sketched a detailed work plan that turned out to be my first thesis draft. The mechanisms we came to as a team (Rennó-Costa et al., 2010, 2014) substantially differed from his initial guesses, but challenging his predictions seemed to excite him rather than provoke frustration.

John frequently expressed his curiosity about brain codes: their form, organization, transmission, and transformations. The theta-gamma code (Lisman and Idiart, 1995; Lisman and Jensen, 2013) is his most prominent work in neural coding and expresses his view on the organization of neural information in the brain. But, he had other contributions. John was enthusiastic about the role of bursts in signal transmission and form. He produced experimental (Erickson et al., 2010) and theoretical (Kepecs et al., 2002; Kepecs and Lisman, 2003) papers that expressed his opinion that bursts could serve both as an information unit but, as well, as a form to pass a signal through. Burst coding was, in part, a reason for his interest in rate remapping. Just before his passing, John was enthusiastic about the role of feedforward inhibition as a high-pass filter in early sensory areas (Yu et al., 2016) and thought that selective bursting could provide a mechanism for reliably transmitting information through neuronal layers in higher hierarchical regions of the brain. This work was left unreleased and is currently under development by collaborators. Other significant contributions of John were a model of competition as code transformation on canonical cortical circuits (de Almeida et al., 2009; Lisman, 2014) and different ideas of how to transform temporal sequences into a spatial code (Sanders et al., 2014).

### Schizophrenia

**Joseph Coyle**, Eben S. Draper Professor of Psychiatry, Harvard University, McLean Hospital, Belmont, MA, USA.

I first got to know John Lisman in 2001. I had organized a Conte Center application for NIMH, focusing on NMDA receptor dysfunction and the hippocampus in schizophrenia. The first submission did not receive a fundable priority, principally because of a weakness concerning the basic neurobiology of the hippocampus. Robbie Green suggested that I contact John, a hippocampal expert, who was located across town at Brandeis. John's initial reaction was that he didn't know anything about schizophrenia and was not interested. However, when he appreciated that he could focus on fundamental aspects of hippocampal physiology on his project, he agreed to participate in the Conte Center proposal, which was ultimately funded for 5 years and re-funded for a second 5-year period.

Soon after the Conte Center was funded, I started to receive calls from schizophrenia experts about John. He was “cold-calling” them and was asking probing questions about schizophrenia, especially with regard to how the hippocampus might be implicated in the disorder. His early studies focused on dopamine interactions in the hippocampus. In a synthetic review, John and Nonna Otmakhova (Lisman and Otmakhova, 2001) demonstrated that dopamine reduces the direct (perforant pathway) cortical input to CA1 but not the CA3 projection. They point out that CA1 plays an important role in novelty detection and under conditions of dopamine hyperfunction or NMDA receptor hypofunction the comparison of endogenous signals from “reality” (cortical) signals would be disrupted, leading to psychotic thinking.

One of the consistent pathologic features in post-mortem studies of schizophrenia is down-regulation of the parvalbumin-positive (PV+), fast-firing GABAergic neurons that provide recurrent inhibition to cortical pyramidal neurons. Collaborating with Margarit Behrens (Zhang et al., 2008), he showed that the pyramidal neurons in adult rats pretreated with the NMDA receptor antagonist, ketamine, exhibited down-regulation of the expression of parvalbumin and glutamic acid decarboxylase 67, the synthetic enzyme of GABA, and disinhibition of the pyramidal neurons. These effects were not observed in immature rats, mirroring the late developmental vulnerability to the psychotomimetic effects of ketamine in humans. Given the critical role of the PV+GABAergic neurons in coordinating pyramidal neuron firing, hypofunction of these neurons could explain the deficits in theta oscillations, which may account for thought disorder and delusional percepts in schizophrenia (Lisman and Buzsáki, 2008).

John organized a symposium for the 2007 annual meeting of the Society for Neuroscience on NMDA receptor dysfunction in schizophrenia and used this as the basis for a grand synthesis: “Circuit-based framework for understanding neurotransmitter and risk gene interactions in schizophrenia” (Lisman et al., 2008). The model linked together the down-regulation of the cortical PV+GABAergic neurons resulting in disinhibition of the pyramidal neurons. This disinhibition decreases the power of gamma oscillations, causing the cognitive and negative symptoms of schizophrenia. This visionary model demonstrated John's incredible ability to synthesize clinical symptoms, neuroanatomy, neurophysiology, genetics and neuropharmacology of schizophrenia. A decade later, this paper still stands as an organizing conceptualization of the disorder that continues to receive more than 50 citations per year.

**Alan Anticevic**, Assistant Professor, Departments of Psychiatry and Psychology, Clinical Neuroscience Research Unit, Yale University School of Medicine, New Haven, CT, USA.

It is my privilege to have this opportunity to honor John's life and his scientific contributions. To put it simply, John Lisman has been an intellectual hero of mine and a remarkably important influence on my career and life. My first introduction to John was through his published work. I distinctly remember, as a first year graduate student, learning about rhythms in the brain from his elegant writing. His ability to articulate and translate his deep insights into accessible concepts has left an indelible impression on me as I was just starting to enter the field. I have never truly developed a deep intuition for how neural oscillations are generated until I was gently but precisely walked through the ideas in one of John's papers. I was hooked.

Years later, as a junior faculty member, I had the opportunity of getting to know John personally while developing a scientific collaboration around our shared interests centered on the thalamus and schizophrenia. I truly cherished our interactions as John challenged me and pushed me to think about the origin of psychosis and the role of distributed cortical and thalamic circuits in the origin of severe mental illness. These exchanges have left a lasting impression and I have come to appreciate the scope and depth of the impact John has generated on countless careers and lives. He was remarkably generous with both his time and his ideas. He always took the time to engage key concepts, debate, teach and, most importantly, provide a unique sense of care and authenticity. One singular trait always came through all of our conversations—a fundamental and infectious curiosity for how the brain works.

On a personal level, John gave me invaluable advice I carry and apply to this day. He sharpened and shaped my thinking. He challenged me. His sense of excitement, optimism and irreverent humor was always contagious. I was lucky enough to see him a few weeks prior to his passing at Woods Hole for a day of scientific discussions with friends and colleagues. As we parted ways, he sharply remarked: “We have unfinished business!,” again referring to our numerous debates about the origins of psychosis and the role of thalamus. His energy, commitment, curiosity and drive to understand the human brain was unique and unrelenting. I miss him deeply.

**Robert P. Vertes**, Professor, Center for Complex Systems and Brain Sciences, Florida Atlantic University, Boca Raton, FL, USA.

It is very sad for me to have to write this in the memory of John. He was such a large presence in life—and in my life. I will forever have lasting memories of John.

I got to know John quite well because for many years, he made yearly trips to South Florida in the spring to visit his in-laws. We would always make it a point to meet, either in my lab at Florida Atlantic University (FAU) or just for lunch, and on a few occasions John gave university-wide seminars. I much looked forward to his visits, for as everyone recognizes, John was a font of knowledge on a wide range of topics, by no means limited to science. And John did not hesitate to express his opinions, particularly on the state of neuroscience and those in the field. It was clear what he liked and disliked, and in a way this was a learning experience for me, as I became more acutely attuned to possible “passing fads” in the field. Since John was so exacting in his own work, it was natural for him to have the highest expectations and hopes for the field. I am a member of the Center for Complex Systems and Brain Sciences at FAU, and a few years ago, we embarked on a review of our program which involved outside consultants. As our Center is an amalgam of various disciplines including basic animal research, modeling and human computational analyses, we needed someone sufficiently adept in each of these areas to serve as a consultant. John was an immediate, consensus candidate. He served in this capacity along with Olaf Sporns of Indiana University. Their recommendations are still a major guiding force for our program.In John's later years he developed an interest in the thalamus, or specifically nucleus reuniens of the ventral midline thalamus—as an integral part of a circuit underlying schizophrenia. As nucleus reuniens is a main interest of mine, this was always one of our topics on his visits. I was gratified by his interest in my work, but more to the point, was awed by how quickly John came to understand this region of the thalamus and its potential impact on the hippocampus and prefrontal cortex in schizophrenia (SZ). In my view, among John's many other accomplishments, his research on thalamocortical circuitry and SZ will lay the groundwork for an eventual full understanding of schizophrenia. I regret that John will not see that day. Obviously, John's passing is a major loss to the field. On a personal level, I have lost a good friend and mentor. I miss you John.

### Large-scale brain integration

**Dan Graboi**, Vice President of Research and Development, VdotP Technologies, Encinitas, CA, USA.

Chance put John and me together as roommates along with George Baral in Rosen Hall in Ridgewood Quadrangle from day 1 at Brandeis in 1962. His interests were in science and politics, photography and girls, and we shared the ups and downs of our intense lives at Brandeis. We moved off-campus our second year to live on the second floor of a house on Fuller Street in Waltham with another Brandeisian, Stephen Altman. During our college years John and I had endless conversations about our greatest shared interest: human perception. There was Minsky's exciting work on the perceptron. Arbib's book—Brains, Machines and Mathematics. Information theory. Exciting concepts of cybernetics and automata. The big question was, how does our brain do it?

As undergraduates, we both took a summer job working in the Brandeis physics lab of Edgar Lipworth. It was a great job. Along with work it included seminars. Imagine! We were paid to learn about atomic beam experiments where time possibly flowed backwards, and quantum physics. During that summer we rented a house in Wellesley with a third housemate. John taught me much about dealing with dishes and cooking. For example, I still put all used dishes and silverware in the sink for later processing, the way he showed me how to do. My wife now does that too. But there was another trick John showed me that I didn't continue: he put together a big pot full of Crisco that would solidify after use, which also held a stainless-steel fry basket. Almost every day that summer, we would pull off the aluminum foil cover, put that pot on the burner, melt the Crisco, and throw in the frozen French fries.

After Brandeis we went in different directions and maintained our friendship. Around the year 2000, John proposed that we collaborate on writing a paper (Graboi and Lisman, 2003). I had no idea what I was letting myself in for! We worked so hard on a publication which described a looping, top-down, bottom-up neural processing algorithm which addressed perception. I cried “Uncle!” many times during the writing of that piece. But John ruthlessly would review every word over and over, questioning everything again and again until finally, he was satisfied. It was an unforgettable experience and high point in my life to work closely with John.

In recent years, we continued to visit each other. John especially enjoyed staying in a wonderful posh Inn in Rancho Santa Fe, where we would meet and always spend much time in the hot tub, and go to some nice restaurant. And as always, much of our conversation would turn to the still mysterious question – how does our brain do it?

The last time I saw John he had brought his family to the Rancho Santa Fe Inn for a brief vacation, and in a caravan of two cars we took an all-day trip into the Anza-Borrego desert. On the road back, John was awestruck by the arrangement of countless boulders strewn over the landscape, left in their places by glaciers in the last ice age.

Who would have thought that fateful day we first met as roommates at Rosen Hall would lead to a lifetime of shared interest and friendship?

**Ivan Soltesz**, James R. Doty Professor of Neurosurgery and Neurosciences, Department of Neurosurgery, Stanford University, Stanford, CA, USA.

To me, John was a true gentle giant of a scholar in the classic sense, someone who just loved thinking about difficult problems, bouncing ideas around, and enjoying the act of scientific communication itself. In addition to the numerous dinner conversations, phone calls and Skype sessions that we had together that are unfortunately largely lost to history forever in terms of their content, I treasure the 450 emails in my inbox that I received from John over the course of a decade and a half. Scrolling through these messages reveals a characteristically Lismanesque treasure-trove of ideas, with the topics ranging from the role of mossy cells in sequence recall (in 2003, way before the recent resurgence of interest in these cells in terms of cognitive processes), the relationship between gamma oscillations and interneurons (2013), the speed of ripple spread in the hippocampus and the conduction velocity of CA3 collaterals (2014) and many others. But, tellingly, the last email from him (on October 7, 2017, less than 2 weeks before his death) was about how we could make sure that his theoretical and computational work continues under our joint BRAIN grant in case something happens to him. His last words were, “I do see this as a 10 year project.” Indeed, his work will continue for a long time in many labs in one form or another precisely because he impacted so many areas of neuroscience and touched so many lives in a positive way in the process.

### A few more personal memories

**Lena Khibnik**, Adjunct Professor, Department of Biological Sciences, North Dakota State University, Fargo, ND, USA.

I joined the Lisman lab as an undergraduate in 2002 to work on my honors thesis. At the time I was relatively new to research, I just knew that I was fascinated with neuroscience and eager to work alongside scientists to learn more about the brain. During my first conversation with John he did not discuss any job responsibilities or expectations, he just took me, extremely enthusiastically, into the world of learning and memory and shared his genuine excitement with me. He posed a question that he thought I could work on—chemical induction of LTP—framing it within the context of current research and sharing with me previous efforts and failures to develop a reliable protocol. At once I felt welcome, challenged, and that I was part of the team. I felt immense gratitude to John because his attitude left no doubt that I could tackle the problem.

I can truly say that despite the fact that I was in the lab for just a short time and only in the very beginning of my journey in research, being part of the Lisman lab was my happiest time in science. I was elated to be doing experiments and absorbing the knowledge though numerous discussions. Hearing John integrate new data into his world of learning and memory was fascinating. But, perhaps, most importantly, I appreciated being heard – I never expected to be given so much autonomy and so much genuine respect as an undergraduate, and the fact that I was taken seriously and regarded as an equal member of the lab was life-changing for me.

John gave me an opportunity and a push to explore the wonders of neuroscience and to grow as a researcher and a critical thinker. He shaped my way of thinking and I continue feeling his impact to this day. John was a brilliant scientist, but also an amazing human being of a rare kind. I am forever grateful that I had the fortune to have him be part of my life.

**Charmian McIntire**, a former graduate student, Lisman lab, Brandeis University, Waltham, MA, USA.

He considered each of us as contributors to something greater than any of us; we were all important - we all had a voice in the Lisman lab. Active regard for any substantiated idea encouraged scientific independence and was a salient hallmark of his mentoring.

**Megan Leubner**, Undergraduate Researcher, Lisman Lab, Brandeis University, Waltham, MA, USA.

Being an undergraduate neuroscience researcher in the Lisman Lab was my first job and Dr. Lisman was my first boss. I learned what it means to be a proper scientist from Dr. Lisman.

I was always in awe of how he never lost sight of the big picture and why we study neuroscience—despite the everyday frustrating minutia that comes with research, he would never give up an opportunity to remind us why we are there. He never thought twice about including us undergraduates in the discussion; we would receive frequent emails about talks and presentations, new papers to review, and fresh questions to investigate. He was remarkable at recognizing talent, promise, and grit, and would challenge us to think critically, engage with the experts, and ultimately grow both individually and as scientists. He was a true mentor in the sense that our successes were his successes. Despite the difference of him having a lifetime of knowledge, he was always confident in our capabilities to fulfill his high expectations. This confidence created a self-fulfilling prophecy—he believed we could, so we did. I felt like a valued member of the team, and I'm incredibly grateful that I was lucky enough to have Dr. Lisman inspire and mentor me.

**Patricia McDonough**, Associate Director, Research Administration, Brandeis University, Waltham, MA, USA.

John Lisman was a passionate scientist with the soul of an artist. He embraced the arts with gusto, appreciating all genres of art: music, theater, painting, and especially photography. In 1994, when the Volen Center for Complex Systems first opened at Brandeis, he saw a blank canvas and strongly felt that science should also be reflected in art form. He took it upon himself and personally installed beautiful scientific photographs throughout the building. To this day, these photographs remain in the hallways of the Volen Center for all to learn, discuss and enjoy. Another one of his personal projects was photographing his neuroscience colleagues. Traveling the world, he went camera in-hand to be sure to garner photographs of colleagues in the various labs that he visited. His goal was to capture the joy and excitement of those scientists who shared the love of understanding the brain. There is one particular memory that stands out in my mind while working for John for over 11 years. One day, while cleaning out his home in preparation to be sold, John brought to the lab a shoebox full of old photos and informed me that these were taken when he was the student photographer as an undergraduate at Brandeis. I could have them if I wanted. No actual work got done that day as I sorted through these amazing photographs of Eleanor Roosevelt, Dr. Martin Luther King, Leonard Bernstein to name a few; photographs of an older Brandeis campus and a young John Lisman. I cannot say for sure that he took all of these amazing pictures, but it was important to protect these photos as part of Brandeis's legacy. I found my way to the University archivist, handed him the shoebox, and explained from whom they came from. The archivist sorted through the photos, and slowly his expression changed from curious to delight and amazement. He concurred that they needed to be archived and sent John a long and grateful thank you letter. Fast forward, these photos have been reproduced and enlarged and are now proudly displayed in various spaces throughout the university campus. When I see them, I smile.

When I think of all of John's contributions to science and the arts, it reminds me how fortunate I have been to be a part of John Lisman's story of life.

**Reza Shadmehr**, Professor of Biomedical Engineering, Professor of Neuroscience, Johns Hopkins University, Baltimore, MD, USA.

Very sad news. Here are a few lines that fit John well. From Late Fragment, a poem by Raymond Carver.

And did you get what you wanted from this life, even so?

I did.

And what did you want?

To call myself beloved, to feel myself beloved on the earth.

## Author contributions

This is a collection of personal tributes to Dr. John Lisman. NAO was one of three organizers of this collection and a main contributor to the Introduction. NO and LG were also organizers of the collection and contributors to the Introduction. They also provided personal tributes in the collection.

### Conflict of interest statement

The authors declare that the research was conducted in the absence of any commercial or financial relationships that could be construed as a potential conflict of interest.
